# ADHD and Moral Development in Childhood and Adolescence: A Systematic Review of Attachment, Temperament, and Socio-Emotional Mechanisms

**DOI:** 10.3390/children13020178

**Published:** 2026-01-28

**Authors:** Ilaria Notaristefano, Federica Gigliotti, Benedetta Altomonte, Ilaria Graziani, Beatrice Piunti, Maria Romani

**Affiliations:** Unit of Child and Adolescent Neuropsychiatry, Department of Human Neuroscience, Sapienza University of Rome, Via dei Sabelli 108, 00185 Rome, Italy; ilaria.notaristefano@uniroma1.it (I.N.); benedetta.altomonte@uniroma1.it (B.A.); ilaria.graziani@uniroma1.it (I.G.); beatrice.piunti@uniroma1.it (B.P.); maria.romani@uniroma1.it (M.R.)

**Keywords:** Attention-Deficit/Hyperactivity Disorder (ADHD), moral development, attachment, temperament, emotion dysregulation, reward sensitivity, callous-unemotional traits, aggression, bullying, social functioning

## Abstract

**Highlights:**

**What are the main findings?**

- Moral development impairments in children and adolescents with ADHD emerge from the interaction of multiple developmental domains, including early attachment insecurity, difficult temperament, emotional dysregulation, altered reward processing, and social dysfunction, rather than from ADHD symptoms alone.
- Across studies, ADHD was consistently associated with delay aversion, reduced fairness and future-oriented decision-making, heightened emotional reactivity and aggression (especially in the presence of CU traits), and increased peer rejection and bullying involvement, all of which compromise moral reasoning, empathy, and prosocial behavior.

**What are the implications of the main findings?**

- Moral development difficulties in ADHD should be conceptualized within a multidimensional developmental framework, highlighting the need for assessment and intervention strategies that go beyond core ADHD symptoms to include emotion regulation, attachment quality, reward sensitivity, and peer relationships.
- Early, developmentally sensitive, and integrative interventions—targeting caregiver–child relationships, emotional self-regulation, social competence, and fairness-related decision-making—may promote both moral growth and long-term socio-emotional outcomes in children and adolescents with ADHD.

**Abstract:**

**Background:** Moral development (MD) arises from the interaction of attachment, temperament, emotion regulation, and decision-making. Children and adolescents with Attention-Deficit/Hyperactivity Disorder (ADHD) frequently show impairments across these domains, suggesting increased vulnerability to disruptions in MD. However, the mechanisms linking ADHD to MD remain poorly understood. **Methods:** A systematic review was conducted according to PRISMA 2020 guidelines. PubMed was searched for studies published between January 2014 and November 2024 examining MD-related constructs, including moral reasoning, fairness, aggression, bullying, callous–unemotional (CU) traits, decision-making, and reward sensitivity, in individuals aged 0–18 years with diagnosed or subclinical ADHD. Due to substantial heterogeneity in study design, measures, and outcomes, a qualitative synthesis was performed. **Results:** Of the 2104 records identified, 23 studies met inclusion criteria. Insecure or disorganized attachment, difficult temperament, and emotional dysregulation consistently emerged as developmental risk factors for impaired MD. Hyperactivity–impulsivity and deficient inhibitory control were strongly associated with aggressive and antisocial behaviors. Children with ADHD demonstrated a pronounced preference for immediate over delayed rewards, altered decision-making in social contexts, and reduced sensitivity to positive feedback. CU traits and aggression were frequently identified as behavioral correlates of MD impairments, particularly in interaction with family adversity and comorbid externalizing conditions. Social dysfunction, including bullying involvement, peer rejection, and interpersonal difficulties, was common and contributed to elevated long-term psychosocial risk. **Conclusions:** ADHD is associated with multidimensional vulnerabilities in MD through intertwined cognitive, emotional, and relational pathways. Interventions targeting attachment security, emotion regulation, reward processing, and social skills may foster MD and reduce later social difficulties. Longitudinal and cross-cultural research is needed to clarify causal mechanisms and inform developmentally sensitive prevention and treatment strategies.

## 1. Introduction

The relationship between moral development (MD) and Attention-Deficit/Hyperactivity Disorder (ADHD) is complex and multidimensional, with significant implications for understanding socio-emotional trajectories in childhood and adolescence. MD begins early in life and is shaped by several interacting factors, including attachment quality, temperament, emotion regulation abilities, and decision-making processes [1,2]. In developmental science, MD is not a unitary construct but a set of partially dissociable processes unfolding across childhood and adolescence. For clarity, we distinguish three complementary domains: (i) moral reasoning/judgment, referring to cognitive evaluations of right–wrong, fairness, intentions, and norm understanding; (ii) moral emotions, including affective dispositions that support concern for others (e.g., empathic concern) and self-evaluative responses (e.g., guilt/shame) that promote reparative behavior; and (iii) moral behavior, namely observable conduct in social contexts, including prosocial actions (helping, sharing, cooperation) and the regulation of aggression, bullying, and rule-breaking. Importantly, constructs such as emotion regulation, reward sensitivity/decision-making, and broader social functioning are discussed in this review primarily as mechanisms and socio-developmental correlates that shape moral outcomes, rather than as synonymous with MD itself [3]. Conceptually, MD can be understood as emerging from the interplay between self-regulatory capacities (e.g., inhibitory control, affect regulation, sensitivity to reward and punishment), social cognitive processes (e.g., interpreting intentions and evaluating fairness), and relational contexts (e.g., caregiver co-regulation and peer inclusion/exclusion) [3,4]. Within this integrative perspective, ADHD-related vulnerabilities may contribute to differentiated moral trajectories not only through core symptoms, but also through associated difficulties in emotion regulation, reward-based decision-making [5,6], and social functioning [4] that shape everyday opportunities for moral learning [1,3]. Among these, early attachment experiences represent a foundational dimension. Attachment refers to a deep and enduring emotional bond connecting individuals across time and space [7]. Classic work by Ainsworth [8] identified four attachment styles—secure, insecure-avoidant, insecure-ambivalent, and disorganized—each associated with different socio-emotional outcomes. Bowlby’s theory posits that early caregiver-child relationships directly influence emotional regulation, social competence, and the development of moral understanding [7]. Wilson [9] further emphasized that a secure attachment base facilitates the natural progression of MD, as children increasingly rely on moral reasoning to navigate social groups and relationships.

Empirical research has consistently documented associations between insecure or disorganized attachment and externalizing behaviors [10,11]. More broadly, insecure attachments are linked to hostility, avoidance, aggression, emotional instability, and anxiety [12]. Difficulties in emotion regulation—a core challenge for many children with ADHD—further amplify these risks. Estimates indicate that 55–75% of children with ADHD exhibit marked emotional dysregulation [13,14]. Conversely, secure attachment promotes emotional regulation, perspective-taking, and the development of a coherent moral framework [3]. Children with disorganized attachment often struggle to implement consistent behavioral strategies in high-stress situations, undermining adaptive moral decision-making [15]. A longitudinal empirical study by Bohlin et al. [16] highlighted strong associations between ADHD symptoms and insecure, particularly disorganized, attachment, partly mediated by deficits in self-regulatory and temperamental functioning. These children may elicit suboptimal caregiving responses, particularly when caregivers have limited tolerance for ADHD-related behaviors [17].

Temperamental self-regulatory capacities emerge early and remain relatively stable across development, shaping the child’s ability to manage emotions and behaviors [18]. Difficult temperament—marked by low adaptability and high negative emotionality—is a well-established risk factor for ADHD symptoms [13]. When combined with deficits in behavioral inhibition, emotional dysregulation may fuel impulsive and aggressive behaviors, particularly under stress. Aggression in children with ADHD can manifest in reactive, emotionally driven responses or in proactive, goal-directed behaviors with minimal emotional involvement, often reinforced through external rewards within peer or familial contexts [19,20]. Such deficits in self-control impede moral and social development and predict adverse academic and occupational outcomes across the lifespan [21,22].

The ability to delay gratification and prioritize long-term benefits represents another key component of MD. From a neurodevelopmental perspective, aberrant reward sensitivity and altered motivational processes have long been implicated in ADHD [5,6]. These alterations contribute to maladaptive decision-making, including risk-taking behaviors, increased accidents, and difficulties in negotiating social interactions [23]. Social dysfunction—characterized by reduced prosocial behaviors and unstable relationships—is a major negative consequence of ADHD and predicts long-term difficulties such as delinquency, anxiety, and broader psychosocial impairments [4].

MD also depends on the accurate perception and interpretation of social cues and on the ability to enact appropriate interpersonal responses. Deficits in these capacities increase the likelihood of confrontational or provocative behaviors, including involvement in bullying. Bullying, defined as repeated intentional aggression toward a weaker individual [24,25], is associated with academic underachievement, substance misuse, aggression, and adverse developmental outcomes [26].

Despite these converging strands of evidence, no systematic review to date has integrated attachment, temperament, emotional dysregulation, reward sensitivity, aggression, and social functioning to clarify how these domains intersect with MD among children and adolescents with ADHD. In particular, prior work has often examined these domains in isolation, leaving unclear how they jointly map onto distinct components of MD (moral reasoning/judgment, moral emotions, and moral behavior) across development in youth with ADHD, and which moderators (e.g., comorbid ODD/CD and family adversity) may account for heterogeneity in outcomes. Clarifying these links is clinically and educationally relevant because “moral difficulties” in ADHD may reflect different underlying pathways that call for different assessment priorities and intervention targets.

The present systematic review therefore aims to (1) synthesize evidence on how ADHD relates to distinct components of MD (moral reasoning/judgment, moral emotions, and moral behavior) across childhood and adolescence; (2) examine how key developmental mechanisms and contexts—attachment, temperament, emotion regulation/irritability, and reward processing/delay-related decision-making—may shape these moral trajectories; and (3) describe behavioral and social correlates (e.g., aggression, bullying involvement, peer difficulties) and major moderators (e.g., comorbid ODD/CD and family adversity) relevant to clinical and educational practice.

## 2. Materials and Methods

### 2.1. Study Design and Reporting

**Figure 1:**
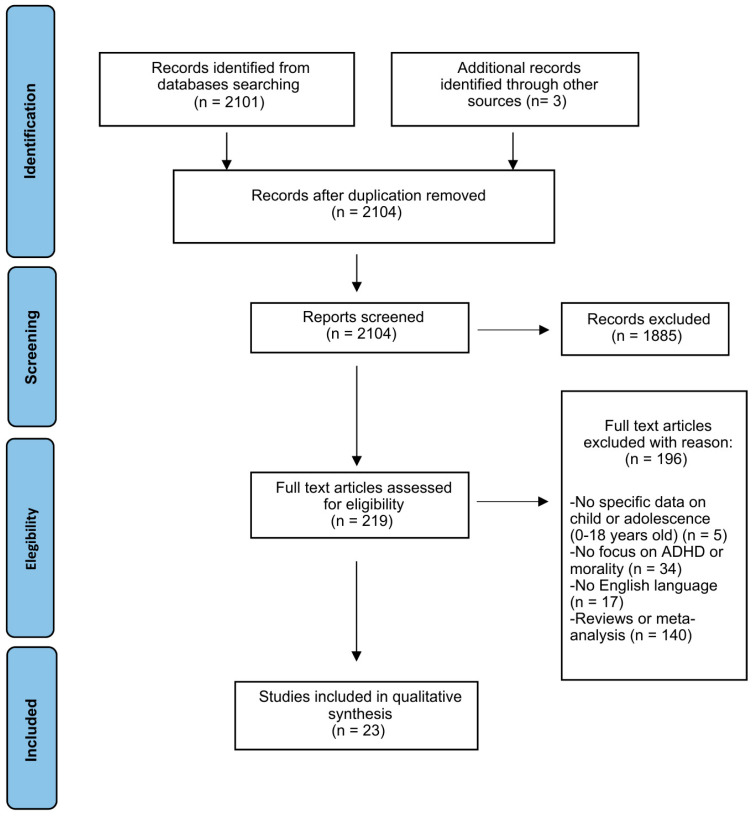
Preferred Reporting Items for Systematic reviews and Meta-Analyses (PRISMA) flow diagram.

This systematic review was conducted in accordance with the Preferred Reporting Items for Systematic Reviews and Meta-Analyses (PRISMA) 2020 guidelines [27]. The PRISMA flowchart summarizing the selection process is presented in Figure 1, and the PRISMA 2020 checklists are provided in the Supplementary Materials (Files S1 and S2). The review protocol was not registered.

### 2.2. Eligibility Criteria

Studies were included if they met the following criteria: (a) original empirical research published in English or with an available English translation; (b) participants aged 0–18 years diagnosed with ADHD or presenting subclinical ADHD symptoms; (c) assessment of MD-related constructs, including moral reasoning, fairness, aggression, bullying, callous–unemotional (CU) traits, decision-making, reward sensitivity, emotion regulation, or social functioning; and (d) quantitative or qualitative empirical designs. Exclusion criteria were (a) studies in which child/adolescent data were not distinguishable from adult samples; (b) studies focusing exclusively on adult populations; (c) studies focusing exclusively on ADHD comorbidities with no MD-related outcomes; and (d) case reports/series, editorials, book chapters, letters, commentaries, and review articles.

### 2.3. Information Sources

A systematic search was performed using the PubMed database to identify studies published between January 2014 and November 2024. Reference lists of previous reviews were also screened. The last search was conducted on 5 November 2024.

### 2.4. Search Strategy

The PubMed search used the following Boolean algorithm: ((ADHD OR attention deficit* hyperactivity disorder*) AND (moral* development* OR fairness OR moral deficit* OR behaviour*) AND (child* OR pediatric* OR kids OR scholar OR adolescence OR adolescent* OR teenager*)). Keywords and MeSH terms were combined to maximize sensitivity. An additional manual search of citations in eligible articles was performed.

### 2.5. Selection Process

Three reviewers independently screened all records in two phases: (1) title and abstract screening and (2) full-text evaluation based on predefined eligibility criteria. Reasons for exclusion at the full-text stage were systematically documented. Discrepancies were resolved through discussion and, when necessary, consultation with a fourth reviewer until consensus was reached. A total of 2104 records were identified; after screening, 23 studies were included.

### 2.6. Data Collection Process and Data Items

Data were extracted independently by three reviewers using a standardized Excel spreadsheet designed according to the PICO framework (Population, Intervention/Exposure, Comparison, Outcome). A fourth reviewer verified all extracted data for accuracy. Extracted variables included: study design, socioeconomic context, sample characteristics (age, gender, size, controls), ADHD diagnostic criteria or measures, constructs related to MD, methods and instruments used, key findings, quality assessment score. Additional information included first author’s name, year of publication, and country of origin.

### 2.7. Quality Assessment

Methodological quality was evaluated using a quality index derived from the Newcastle–Ottawa Scale (NOS) adapted to cross-sectional and longitudinal designs [28]. Each study was assigned a score based on selection, comparability, and outcome domains. Full details are provided in Supplementary Tables S1 and S2.

### 2.8. Synthesis Methods

Due to substantial heterogeneity across study designs, populations, and outcome measures, a qualitative thematic synthesis was performed. Studies were grouped into conceptual domains relevant to MD processes: (1) attachment and temperament; (2) emotion regulation and aggression; (3) CU traits; (4) reward sensitivity and decision-making; and (5) social functioning and bullying. Patterns were summarized, and explanatory mechanisms were explored.

## 3. Results

Figure 1 presents the PRISMA flow diagram. A total of 23 studies met the inclusion criteria and were included in the final synthesis. To facilitate interpretation, findings are discussed in relation to the three MD components (moral reasoning/judgment, moral emotions, moral behavior), while results are organized into thematic developmental domains (attachment/temperament, reward-based decision-making, emotion dysregulation/CU traits, and social functioning/bullying) hypothesized to shape moral outcomes, which are discussed in this review as developmental mechanisms/correlates rather than MD components per se. Table 1 summarizes the characteristics of the included studies, while Supplementary Tables S1 and S2 report the quality assessment.

### 3.1. General Characteristics of the Studies

Quality scores ranged from 4 to 7 out of 9 on the adapted Newcastle–Ottawa Scale, with a mean of 6.21 and a median of 6, indicating moderate methodological quality [52]. Twelve studies adopted a longitudinal design, while eleven were cross-sectional; among these, five used a case–control approach. Sample sizes ranged from 57 to 125,621 participants, with ages spanning 2–18 years. In total, the review included data from 146,314 individuals across fifteen countries. Most studies were conducted in Europe (17 studies; Netherlands, United Kingdom, Finland, Sweden, Portugal, Switzerland, Belgium). Others were carried out in the United States (3), China (1), Thailand (1), Malaysia (1), and Canada (1). Socioeconomic context was assessed in only five studies, and inconsistently across them. Studies were grouped according to the main constructs examined and the measures employed (see Table 1).

### 3.2. Attachment and Temperament as Early Foundations of Moral Development

Two studies examined early relational and temperamental precursors of MD. Forslund et al. [32] and Morales et al. [37] found that disorganized attachment was associated with conduct problems, but not directly with ADHD symptoms. Building on this developmental perspective, Wu et al. [50] examined the long-term influence of early temperament. Difficult temperament at age 2 predicted a higher likelihood of becoming NEET (Not in Education, Employment, or Training) in adulthood. Hyperactivity-impulsivity symptoms mediated the pathway from difficult temperament to NEET status, whereas inattention did not. Adolescent antisocial behaviors further contributed to this developmental trajectory [50]. In adolescence, temperament profiles have also been associated with socio-emotional functioning relevant to moral behavior. In a cross-sectional study, Deotto et al. [31] reported that high harm avoidance and low self-directedness were associated with internalizing symptoms in adolescents with ADHD, whereas low cooperativeness was linked to externalizing behaviors, independently of ADHD severity and working memory performance.

### 3.3. Reward Anticipation and Decision-Making in ADHD

Five studies focused on reward processing and decision-making. Across all studies, children and adolescents with ADHD consistently preferred immediate, smaller rewards over delayed, larger ones. Using the Ultimatum and Dictator Games, Ma et al. [34] found that individuals with ADHD made lower and more self-serving offers than typically developing peers, prioritizing personal gain over fairness. Northover et al. [38] reported that youth with ADHD and comorbid Conduct Disorder were more likely to reject moderately unfair or ambiguous offers, reflecting heightened emotional reactivity. Mies et al. [36] observed increased impatience and lower tolerance for delay in gain-related conditions among children with ADHD. Van Dessel et al. [47] expanded on these findings at the neural level using functional MRI: although overall activation during monetary loss processing did not differ between groups, controls showed greater ventral striatum activity during loss avoidance. By contrast, ADHD participants exhibited reduced sensitivity to positive feedback and increased anterior insula activation to negative feedback. Kostyrka-Allchorne et al. [33] demonstrated that heightened preference for immediacy co-occurred with reduced capacity for future-oriented thinking. Importantly, impaired simulation of future rewards did not mitigate delay aversion. These deficits remained significant after adjusting for aggression [33].

### 3.4. Emotional Dysregulation, Aggression, and Moral Challenges

Nine studies examined emotional dysregulation and its association with aggressive and disruptive behaviors. Waller et al. [48] and Speyer et al. [43] emphasized that aggression in ADHD is heterogeneous, with distinct reactive and proactive components associated with divergent developmental risks, including academic failure, peer rejection, and involvement in the juvenile justice system. Marques et al. [35] identified inhibitory control deficits as contributors to emotional dysregulation, which mediated the link between ADHD symptoms and aggression. Colonna et al. [30] reported strong associations between irritability—a core component of emotional dysregulation—and executive dysfunction. Several studies investigated CU traits. Zhang et al. [51] found that CU traits in Chinese preschoolers with ADHD predicted more severe behavioral problems. Similarly, Waller et al. [48] identified CU traits and oppositional behaviors as markers of early risk for conduct problems in ADHD. Thorell et al. [45] showed that both emotional dysregulation and ADHD symptoms predicted peer rejection and difficulties with cooperative interactions over time. Tengsujaritkul et al. [46] highlighted the broader academic and social impairments associated with emotional and behavioral difficulties in ADHD.

### 3.5. Social Functioning and Bullying

Three studies reported clear associations between ADHD and increased aggression. Bartels et al. [29] showed age-dependent patterns, with ADHD more strongly associated with physical aggression in childhood and rule-breaking behaviors in adolescence. Studies from the Swiss Z-proso project found developmental continuity: Speyer et al. [44] reported that ADHD symptoms at age 7 predicted externalizing behaviors at age 9, which in turn predicted a resurgence of ADHD symptoms at age 11. Ribeaud et al. [41] documented persistent ADHD symptoms from ages 7–17 predicting long-term externalizing behaviors. However, not all findings were consistent. Ramsey et al. [40] observed only a marginal association between ADHD symptoms and rule-breaking, and no significant link with aggression; instead, aggression was more strongly associated with anger. Broader social outcomes were addressed by Savolainen et al. [42], who reported links between ADHD, academic difficulties, peer marginalization, and alcohol use. Notably, ADHD—but not Conduct Disorder—was uniquely related to peer marginalization. Wan Ismail et al. [49] found that social isolation associated with ADHD reduced opportunities for bullying perpetration. In contrast, hyperactive and inattentive symptoms individually increased the likelihood of bullying behaviors, whereas the combined presence of inattention and hyperactivity showed a protective association. The authors also identified oppositional defiant disorder (ODD) and conduct disorder (CD) as mediating and moderating factors in the persistence of aggressive and bullying behaviors. Rajendran et al. [39] similarly reported higher bullying involvement among children with ADHD, both with and without ODD, and identified parenting practices—specifically negative affectivity and promotion of excessive autonomy—as predictors of bullying.

### 3.6. Overall Synthesis

Taken together, findings across the 23 included studies indicate that children and adolescents with ADHD present multidimensional vulnerabilities in MD. Early attachment and temperament contribute to later socio-emotional outcomes; impairments in reward processing and future-oriented thinking affect fairness and decision-making; emotional dysregulation and CU traits shape aggressive behavior; and social dysfunction influences involvement in bullying and long-term maladaptive trajectories.

## 4. Discussion

This systematic review synthesizes evidence from 23 studies investigating how core developmental domains—attachment, temperament, emotion regulation, reward processing, and social functioning—intersect with MD in children and adolescents with ADHD. Across the included studies, diverse patterns emerged that point to multiple potential mechanisms connecting ADHD-related characteristics with aspects of MD. These findings offer a foundation for interpreting how developmental and socio-relational processes may influence moral reasoning, fairness, empathy, and prosocial behavior in youth with ADHD. Given the heterogeneity of study designs and the predominance of cross-sectional evidence, the associations discussed below should not be interpreted as causal pathways. Rather, we integrate findings across domains as a qualitative framework to highlight converging mechanisms and sources of heterogeneity in socio-moral outcomes among youth with ADHD.

### 4.1. Attachment, Temperament, and Early Relational Foundations of Moral Development

Across the included studies, insecure or disorganized attachment emerged as an early relational risk factor for socio-behavioral outcomes relevant to MD. Although disorganized attachment was associated with both ADHD symptoms and conduct problems, findings consistently showed that its independent association was stronger for conduct-related outcomes than for ADHD symptoms per se. In this regard, the authors suggested that the apparent association between disorganized attachment and ADHD may be partly explained by the high comorbidity between ADHD and conduct disorders. In those studies, conduct problems were reported to be more closely related to negative emotionality and inconsistent stress responses typically observed in disorganized attachment, whereas ADHD symptoms were more closely related to inhibitory control difficulties and regulation of positive emotions [32,37]. These results also align with broader literature suggesting that apparent attachment disorganization in ADHD may reflect difficulties in behavioral and emotional self-regulation—sometimes described as “pseudo-disorganization”—rather than true attachment system disruption [32]. From a clinical standpoint, these findings are consistent with the possibility of differentiated pathways to moral difficulties among youth with ADHD. In a relational-adversity pathway, disorganized attachment may index early caregiving stress and inconsistent co-regulation and appears more proximally linked to conduct-related/externalizing outcomes than to ADHD symptoms per se [32,37]. In parallel, a neurodevelopmental self-regulatory pathway—involving inhibitory control, reward-based learning/delay-related decision-making, and emotion regulation—may shape day-to-day moral decision-making and behavior in ADHD without necessarily implying attachment system disruption [5,6,14]. Importantly, these pathways may co-occur and interact in clinical presentations, underscoring the need to interpret moral difficulties in ADHD as potentially arising from distinct—yet converging—relational and self-regulatory mechanisms. Secure attachment, conversely, supports emotional regulation and perspective-taking [3], capacities essential to the development of empathy and moral reasoning. Temperamental factors added an additional layer of early vulnerability. Difficult temperament in toddlerhood predicted long-term negative outcomes [50], with hyperactivity-impulsivity mediating pathways from early negative emotionality to adolescent antisocial behavior. These findings complement theoretical models that conceptualize temperament as an early-developing regulatory system shaping emotional reactivity and self-control [18]. In this context, high reactivity and low effortful control may undermine early moral learning by increasing frustration, reducing compliance, and heightening conflictual interactions with caregivers.

### 4.2. Reward Processing, Delay Aversion, and Fairness-Related Decision-Making

A consistent theme across studies was altered reward processing in ADHD. Children with ADHD demonstrated a pronounced preference for smaller immediate rewards over larger delayed ones [33,36], particularly in gain- oriented contexts. These preferences mirror well-established motivational alterations in ADHD, including heightened reward sensitivity and difficulty sustaining motivation across delays [5,6]. Neuroimaging findings further showed reduced ventral striatum activation during reward anticipation and heightened anterior insula activation to negative feedback [47], suggesting altered integration of reward value and feedback valence. Such abnormalities have direct implications for MD. In fairness-related tasks, including the Ultimatum and Dictator Games, children with ADHD made more self-serving offers or showed heightened rejection of ambiguous offers [34,38]. Reduced responsiveness to social rewards compared to monetary ones [53] may undermine cooperative behaviors and limit opportunities to internalize social norms. Ultimately, difficulties evaluating future consequences and balancing self-interest with fairness can compromise moral judgment and prosocial decision-making.

### 4.3. Emotional Dysregulation, Irritability, and Moral Competence

Emotional dysregulation emerged as a central mechanism linking ADHD to aggression, irritability, and interpersonal difficulties. Several studies highlighted associations between inhibitory control deficits, frustration intolerance, and aggressive behavior [30,35]. These findings converge with broader evidence that 55–75% of children with ADHD experience significant emotional dysregulation [14], and that irritability represents a critical affective dimension contributing to reactive aggression [54]. Such emotional vulnerabilities can disrupt moral learning by impairing children’s ability to consider others’ perspectives, regulate anger, or resolve conflicts constructively. Social consequences of emotional dysregulation were also evident. Studies consistently showed that ADHD symptoms and emotional difficulties predicted peer rejection, unstable friendships, and challenges in cooperative play [43,45,48]. These relational difficulties reduce access to positive peer models and prosocial feedback, potentially undermining opportunities to develop empathy reciprocity, and moral understanding. CU traits represented an especially high-risk profile. Children with ADHD and elevated CU traits showed more severe and persistent aggression [48,51]. Reduced guilt, diminished empathic concern, and insensitivity to punishment—features characteristic of CU traits—present additional obstacles for MD and may require more targeted intervention, particularly in cross-cultural contexts.

### 4.4. Social Functioning, Peer Dynamics, and Bullying Involvement

Social functioning emerged as a pivotal domain through which ADHD affects MD. Hyperactivity, inattention, and emotional dysregulation predicted increased aggression, rule-breaking, and externalizing behaviors [29,41,44]. Comorbid ODD and CD further heightened risks for bullying involvement [39].

Peer marginalization played a particularly important role. ADHD symptoms—independent of conduct problems—were associated with peer rejection and social exclusion [42], consistent with evidence that ADHD is linked to reduced prosocial behaviors and unstable peer relationships [4]. These experiences may hinder moral learning by limiting exposure to cooperative interactions and opportunities to practice moral reasoning in dyadic and group settings. Bullying involvement followed complex patterns. Children with ADHD were more likely to be perpetrators, victims, or bully victims [49], with gender differences suggesting that boys tend to show more perpetration, while girls are more frequently victimized. Additional risk factors—including emotional dysregulation, academic challenges, and socioeconomic adversity—further contributed to bullying dynamics. Behavioral and social impairments also extended to academic contexts, with emotional and behavioral dysregulation negatively affecting school engagement and performance [46]. These patterns underscore how ADHD-related difficulties in cognition, emotion, and social interaction jointly shape MD-relevant outcomes.

### 4.5. Integrative Interpretation Across Domains

Overall, findings from the 23 included studies converge on a multidimensional developmental model. Early attachment insecurity and difficult temperament disrupt emotional regulation and behavioral control. Altered reward processing biases children toward immediate gratification and reduces sensitivity to fairness and social incentives. Emotional dysregulation and CU traits intensify aggressive tendencies and diminish empathy. Peer rejection, social exclusion, and bullying involvement limit opportunities for positive social learning. Importantly, across studies, moral behavioral outcomes (e.g., aggression, rule-breaking, bullying involvement) were most consistently amplified in the presence of key moderators such as comorbid ODD/CD and family adversity (e.g., chronic stress and socioeconomic disadvantage), which likely contribute to heterogeneity in “moral difficulties” observed in ADHD. These interconnected domains suggest that socio-moral functioning in ADHD can be understood as emerging from the interplay between self-regulatory capacities (e.g., inhibitory control, affect regulation, delay-related decision-making), social cognitive processes (e.g., intention understanding, fairness evaluation), and relational contexts (e.g., caregiver co-regulation and peer inclusion/exclusion). The empirical evidence synthesized here supports consistent associations across these domains; however, the proposed pathways should be considered a qualitative, hypothesis-generating framework rather than a causal account, and require longitudinal testing to establish temporal ordering and mechanisms.

**Figure 2:**
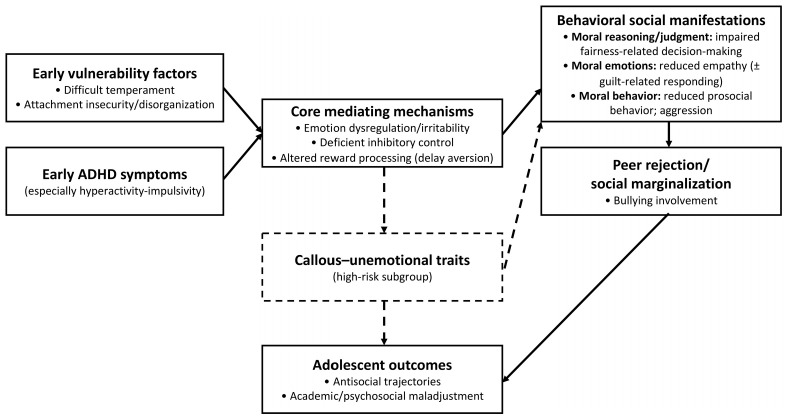
Intertwined developmental pathways linking ADHD to MD difficulties. Dashed arrows indicate hypothesized pathways relevant to a high-risk subgroup (callous–unemotional traits).

To summarize the qualitative synthesis and provide an integrative framework for the intertwined pathways linking ADHD to MD difficulties across domains, we present a conceptual flowchart in Figure 2.

### 4.6. Clinical and Educational Implications

The present synthesis suggests that socio-moral difficulties observed in youth with ADHD should be assessed beyond core symptoms, by systematically considering key moderators and targets such as comorbid ODD/CD and callous–unemotional traits, family adversity and caregiving stress, emotion dysregulation/irritability, reward-based decision-making (delay aversion), and peer functioning (peer rejection and bullying involvement). Clinically, these domains may help differentiate heterogeneous developmental pathways and refine case formulation and intervention priorities. In practice, interventions may be most effective when they integrate established ADHD care (behavioral and educational supports, with pharmacotherapy when indicated) with components targeting emotion regulation and social decision-making in everyday contexts [55]. When peer dynamics and bullying are prominent, coordinated, whole-school preventive approaches may be warranted alongside clinical care [56].

### 4.7. Limitations and Future Directions

Several limitations should be considered when interpreting the findings of this review. First, the search strategy was restricted to PubMed and the review protocol was not preregistered (e.g., PROSPERO); therefore, relevant studies indexed primarily in other databases may have been missed, selective reporting cannot be fully excluded, and publication bias cannot be ruled out. Second, the included studies exhibited substantial methodological heterogeneity in terms of design, measures of MD, and assessment of ADHD-related characteristics. This variability limited direct comparisons and precluded the possibility of conducting a quantitative meta-analysis, so conclusions should be interpreted as a qualitative, hypothesis-generating synthesis rather than as precise pooled estimates. Third, we did not conduct a formal assessment of the overall certainty of the body of evidence (e.g., GRADE), which further supports cautious interpretation of the strength of the inferences. Fourth, the geographic distribution of the studies was uneven, with most research conducted in Europe and North America, limiting the cultural generalizability of the findings. Fifth, many studies did not comprehensively address comorbidities such as ODD, CD, or internalizing symptoms, despite their known influence on socio-emotional functioning and MD. Finally, most evidence was cross-sectional or based on short-term longitudinal designs, restricting the ability to draw causal inferences about developmental trajectories. Several avenues for future research emerge from these limitations. Expanding investigations to underrepresented regions and cultural contexts is essential for improving ecological validity and capturing culturally specific pathways to MD. More rigorous attention to comorbidity would allow for a clearer understanding of how overlapping conditions shape MD in ADHD. Longitudinal studies spanning childhood through adolescence are particularly needed to clarify causal mechanisms and identify sensitive developmental periods for intervention. Multidisciplinary approaches integrating developmental psychology, education, and neurobiological methods—including advanced neuroimaging and computational models—may elucidate the neural and cognitive bases of moral reasoning difficulties in ADHD. Additionally, refinement of experimental paradigms assessing reward sensitivity, social decision-making, and moral judgment would deepen our understanding of the specific processes involved. Finally, designing and evaluating targeted interventions that strengthen protective factors—such as secure attachment, emotion regulation skills, and social competence—represents a promising direction for supporting moral and socio-emotional development in children and adolescents with ADHD.

## 5. Conclusions

This systematic review highlights the multifaceted relationship between ADHD and MD, showing that ADHD extends beyond core neurodevelopmental symptoms to involve significant emotional, cognitive, and socio-relational dimensions relevant to moral functioning. Across the reviewed studies, difficulties in emotion regulation, impulse control, reward processing, and social interactions emerged as central mechanisms that may hinder the acquisition of moral competencies in children and adolescents with ADHD. By identifying key developmental pathways—from early attachment insecurity and difficult temperament to altered decision-making and peer-related challenges—this review provides a conceptual framework for understanding how ADHD intersects with moral reasoning, fairness, empathy, and prosocial behavior. These insights underscore the need for holistic and developmentally sensitive approaches in both clinical and educational settings. Supporting emotional regulation, strengthening caregiver–child relationships, and promoting positive peer experiences may contribute not only to improved behavioral regulation but also to the cultivation of moral growth and social integration in youth with ADHD. Taken together, these findings support assessment and intervention beyond core ADHD symptoms—toward emotion regulation/irritability, attachment security and caregiving stress, sensitivity to social reward and fairness-related decision-making, and broader socio-moral competencies relevant to peer functioning—while underscoring the need for longitudinal, cross-cultural, multi-method studies with rigorous control of key moderators to support causal conclusions.

## Figures and Tables

**Table 1:** General characteristics of the studies.

Author (Year)	Country	Design	Sample (N, %M)	Age Range (Years)	Tools	Outcome	Main Findings	Limitations
Bartels M. et al. (2018) [29]	Netherlands, United Kingdom, Finland, Sweden	L	125,621 (49%)	3–16	CBCL, YSR, DCB, SDQ, A-TAC, MPNI	Co-occurrence of aggression and childhood psychopathology	Aggression co-occurred with attention, internalizing, and oppositional problems across development, with decreasing gender differences over age	Cross-national heterogeneity; observational design; multiple measurement tools
Colonna S. et al. (2022) [30]	Wales	L	219 (94%)	10–18	CAPA, ChATTI, CRS (teacher form), DAWBA, WCST, GnG, CPT, TDT, UG, CxR, SES (parental occupation), WASI (vocabulary, matrix reasoning)	Cognitive mechanisms of irritability in ADHD (hot vs. cool executive functions)	Irritability in youths with ADHD was not associated with reward-processing deficits, despite extensive assessment of hot and cool executive functions	Observational design; limited transdiagnostic scope; need for replication
Deotto A. et al. (2022) [31]	Canada	Cx	121 (74%)	13–18	K-SADS, SWAN, SDQ, TCI, WISC-IV	Temperament factors associated with internalizing and externalizing symptoms in ADHD	Distinct temperament profiles were identified in adolescents with ADHD: harm avoidance predicted internalizing symptoms, low cooperativeness predicted externalizing behaviors, and inattentive symptoms were directly linked to aggression	Cross-sectional design; unmeasured transdiagnostic constructs; limited causal inference
Forslund T. et al. (2016) [32]	Sweden	Cx	184 (49%)	6–7	Emotion Questionnaire, ADHD Rating Scale-IV, SDQ (parent report), SAT, Stroop (Day-Night), GnG	Associations between ADHD symptoms, cognitive inhibition, and positive emotion regulation	ADHD and conduct problems followed distinct developmental pathways: ADHD was associated with inhibition deficits and poor positive emotion regulation, whereas conduct problems were linked to disorganized attachment and high negative emotionality	Parent-report bias; non-clinical sample; low symptom prevalence
Kostyrka-Allchorne K. et al. (2020) [33]	United Kingdom	Cx	64 (44%)	11–17	AFTA, QDQ, CFC, CRS-3 (parent short form), WASI-II, Temporal Discounting Task.	Prospection, delay discounting, and time-oriented decision-making in adolescents with ADHD	ADHD symptoms were associated with reduced prospection and preference for future rewards; both constructs were independent and unrelated to aggression	Small sample size; subjective assessment of prospection; low aggression levels
Ma I. et al. (2017) [34]	Netherlands	Cx-CC	67 (60%)	9–17	UG, DG, DISC-IV, IRI	Decision-making processes in youth with ADHD (economic games)	Youth with ADHD showed more self-interested and reward-driven decision-making in social economic games, despite intact empathy and perspective-taking	Small sample size; limited reward sensitivity measures; lack of ecological social measures
Marques S. et al. (2024) [35]	Portugal	Cx	72 (64%)	6–10	CRS (parent short form); BRIEF (parent form), CBCL, ERC	Mediating role of emotion dysregulation and depressive symptoms in the link between inhibitory control and aggression in ADHD	Emotion dysregulation and depressive symptoms mediated the association between inhibitory control deficits and aggressive behavior in children with ADHD	Single-informant reports; convenience sampling; cross-sectional design
Mies G.W. et al. (2019) [36]	Netherlands	Cx-CC	57 (37%)	12–17	Delay discounting tasks, QDQ, BIS/BAS, BIS-11, SHAPS, SRS	Immediate versus delayed reward decision-making in adolescents with ADHD	Adolescents with ADHD showed greater delay aversion, contributing to impulsive decision-making and reduced long-term planning	Small sample size; task familiarity effects; unmeasured confounders
Morales M.F. et al. (2023) [37]	Scotland	L	3578 (51%)	4–10	SDQ, DASS, MPAS, BAS-II, SIMD	Developmental trajectories, co-occurrence, and multimorbidity of hyperactivity/inattention, conduct problems, and peer problems, including risk and protective factors	Six developmental trajectories of conduct problems, hyperactivity/inattention, and peer problems were identified, with attachment quality and caregiver mental health acting as protective factors	Parent-report bias; psychometric limitations; underrepresentation of high-risk families; no causal inference; short follow-up
Northover C. et al. (2015) [38]	United Kingdom	Cx-CC	231 (100%)	10–18	DAWBA, SDQ, YPI, UG, WASI	Emotion regulation and decision-making in boys with ADHD, with and without conduct disorder	Boys with ADHD and high aggressive conduct symptoms showed atypical rejection of ambiguous offers, indicating impaired emotion regulation and heterogeneity within ADHD	Male-only sample; lack of emotional response measures; limited ecological validity
Rajendran K. et al. (2016) [39]	United States	L	162 (71%)	4–9	Nakao and Treas Socioeconomic Prestige Index, NICHD Coding System for Mother–Child Interactions, K-SADS, teacher-reported bullying assessment	Bullying behaviors in ADHD and ADHD+ODD and the role of parenting	Children with comorbid ADHD and ODD showed higher bullying involvement, whereas early parental support for autonomy was associated with reduced bullying	Limited bullying assessment; single-parent informant; no victimization analysis
Ramsey K.L. et al. (2022) [40]	United States	Cx	119 (100%)	14–18	APS-SF, STAXI-2 C/A, staff-reported behavioral records	Associations between ADHD symptoms and misconduct in juvenile justice adolescents	Trait anger predicted disruptive and rule-violating behaviors, while ADHD symptoms showed only marginal associations with institutional misconduct	Institutionalized sample; archival and self-report data; limited generalizability
Ribeaud D. et al. (2022) [41]	Switzerland	L	1239 (52%)	7 at baseline	SBQ, APQ, standardized questionnaires assessing mental health, aggression, ADHD symptoms, substance use, and victimization	Social development and prevention of aggressive and antisocial behavior (parenting and socio-emotional interventions)	The PATHS intervention reduced teacher-rated externalizing behaviors at age 11; alcohol use and low academic performance were associated with increased behavioral risk, and developmental cascades linked early ADHD symptoms to later aggression and internalizing problems, while sensation-seeking predicted late-onset ADHD	Longitudinal attrition; socioeconomic sample bias; limited rare-outcome detection
Savolainen J. et al. (2015) [42]	Finland	L	4644 (100%)	15 at baseline	YSR, data collected from The Central Register for Criminal Records and from Finnish Health Care Registry for childhood diagnoses	Pathways linking ADHD to felony offending (substance use, school failure, peer influences)	Alcohol use and low academic achievement increased the risk of felony conviction; ADHD was associated with peer marginalization, whereas peer marginalization itself did not predict offending	Male-only sample; unclassified ADHD subtypes; survey attrition
Speyer L.G., Eisner M. et al. (2022) [43]	Switzerland	L	1246 (51%)	7–11	SBQ	Bidirectional associations between ADHD symptoms and aggressive behaviors	Inattentive symptoms showed reciprocal longitudinal associations with both proactive and reactive aggression, suggesting a central role in aggressive development	Limited symptom indicators; low statistical power; community-based sample
Speyer L.G., Obsuth I. et al. (2022) [44]	Switzerland	L	1387 (60%)	7–11	SBQ, APQ, teacher-reported academic achievement and peer problems	Developmental cascades linking ADHD, internalizing and externalizing symptoms (parenting and peer factors)	No significant mediating mechanisms were identified in within-person developmental cascades linking ADHD, internalizing, and externalizing symptoms	Non-robust cross-informant effects; limited measure specificity
Thorell L.B. et al. (2017) [45]	Sweden	L	91 (39%)	6–9.5	CRS-3 (short form), Emotion Questionnaire	Early ADHD symptoms and emotional functioning as predictors of later peer problems	Early ADHD symptoms and emotion dysregulation (especially happiness/exuberance) predicted later peer rejection and physical aggression	Small non-clinical sample; parent-report only; limited generalizability
Tengsujaritkul M. et al. (2020) [46]	Thailand	Cx-CC	80 (83%)	6–10	SNAP-IV, CBCL, SDQ	Emotional/behavioral problems and functional impairment in children with ADHD versus controls	Children with ADHD showed persistent emotional, behavioral, and social impairments despite treatment, particularly in the combined subtype	Small clinical sample; parent-report only; treatment details not analyzed
Van Dessel J. et al. (2022) [47]	Belgium	L-CC	66 (100%)	8–18	EMLI task under functional magnetic resonance imaging, valence and motivation rating scales	Neural and behavioral responses to monetary loss and reinforcement in ADHD	Youth with ADHD showed altered neural responses to reward outcomes, including reduced ventral striatum activation to success and increased insula activation to failure	Male-only sample; task training effects; pubertal development not controlled
Waller R. et al. (2015) [48]	United States	L	240 (51%)	3–6	CBCL, Inventory of Peer Relations, CBQ, ‘My Child’ questionnaire, False Belief Prediction and Explanation Tasks-Revised, Kochanska et al.’s toddler-aged battery, Denham’s emotion understanding tasks	Developmental pathways to conduct problems	ADHD and oppositional behaviors were associated with impulsivity and emotion regulation difficulties, whereas CU traits were characterized by reduced empathy and moral engagement and were linked to more severe and proactive antisocial behaviors.	Community sample bias; shared method variance; limited behavioral specificity of CBCL
Wan Ismail W.S. et al. (2014) [49]	Malaysia	Cx	410 (48.7%)	12	MBQ, CASS:S, CPRS:S, CTRS:S, CBCL	Sociodemographic and psychological correlates of bullying behavior	Male sex, ADHD symptoms, and conduct problems increased bullying risk, whereas internalizing symptoms were protective	Cross-sectional design; self-reported bullying; limited causal inference
Wu T.C.H. et al. (2022) [50]	England	L	6240 (39%)	2 at baseline	NEET status self-report, CTTS, DAWBA; antisocial behaviour self-report	Toddler temperament as a predictor of NEET status in adulthood	Difficult temperament in toddlerhood predicted NEET status in adulthood via hyperactivity–impulsivity and later antisocial behavior	Parent-report bias; self-reported antisocial behavior; limited causal inference; limited generalizability
Zhang J. et al. (2021) [51]	China	Cx	176 (81%)	4–5.11	SNAP-IV, ICU, BRIEF preschool version, SDQ	CU traits in preschool children with ADHD and ODD	Preschool children with ADHD showed elevated CU traits associated with executive function deficits and increased risk of ODD/CD	Cross-sectional design; no CD/ODD subgrouping; limited developmental inference

L = longitudinal studies; Cx = cross-sectional studies; CC = case–control studies; N = total sample size; %M = percentage of males; CBCL = Child Behaviour Checklist; YSR = Youth Self-Report; DCB = Devereux Child Behaviour; SDQ = The Strengths and Difficulties Questionnaire; A-TAC = The Autism–Tics; MPNI = Multidimensional Peer Nomination Inventory; CAPA = Child and Adolescent Psychiatric Assessment; ChATTI = Child ADHD Teacher Telephone Interview; CRS = Conners Rating Scale; DAWBA = Development and Well Being Assessment; WCST = Wisconsin Card Sorting Test; GnG = “Go no Go” task; CPT = Card Playing Task; TDT = Temporal Discounting Task; UG = Ultimatum Game; CxR = Choice per Risk Task; SES = Socioeconomic Status; WASI = Wechsler Abbreviated Scale of Intelligence; K-SADS = Schedule for Affective Disorders and Schizophrenia for School-Age Children; SWAN = Strengths and Weaknesses of ADHD-symptoms and Normal-behaviors rating scale; TCI = Temperament and Character Inventory; WISC-IV = Wechsler Intelligence Scale for Children, Fourth Edition; SAT = Separation Anxiety Test; AFTA = Adolescent Future Thinking Assessment; QDQ = Quick Delay Questionnaire; CFC = Consideration of Future Consequences questionnaire; WASI-II = Wechsler Abbreviated Scale of Intelligence, Second Edition; DG = Dictator Game; DISC-IV = Diagnostic Interview Schedule for Children; IRI = Interpersonal Reactivity Index; BRIEF = Behavior Rating Inventory of Executive Function; ERC = Emotion Regulation Checklist; BIS/BAS = Behavioral Inhibition/Approach System Scales; BIS-11 = Barratt Impulsiveness Scale; SHAPS = Snaith–Hamilton Pleasure Scale; SRS = Social Responsiveness Scale; DASS = Depression Anxiety Stress Scales; MPAS = Maternal Postnatal Attachment Scale; BAS-II = British Ability Scales; SIMD = Scottish Index of Multiple Deprivation; YPI = Youth Psychopathic Traits Inventory; NICHD = National Institute of Child Health and Human Development; APS-SF = Adolescent Psychopathology Scale—Short Form; STAXI-2 C/A = State Trait Anger Expression Inventory Child and Adolescent; ADHD = Attention-Deficit/Hyperactivity Disorder; SBQ = Social Behavior Questionnaire; APQ = Alabama Parenting Questionnaire; SNAP-IV = Swanson, Nolan, and Pelham version IV Scale; EMLI = Escape Monetary Loss Incentive; CBQ = Children’s Behavior Questionnaire; MBQ = Malaysian Bullying Questionnaire; CASS:S = Conners-Wells Self-Report: Short Form; CPRS:S = Conners’ Parents Rating Scale: Short Form; CTRS:S = Conners Teachers Rating Scale: Short Form; NEET = Not in education, employment or training; CTTS = Carey Toddler Temperament Scale; ICU = Inventory of Callous–Unemotional Traits; ODD = oppositional defiant disorder; CU = callous–unemotional; PATHS = Promoting Alternative Thinking Strategies; CD = conduct disorder.

## Data Availability

No new data were generated in this study. Data extraction forms/coding sheets supporting the conclusions of this article are available from the authors upon reasonable request. They are not publicly available because they are derived from third-party published sources and form part of the authors’ working documents.

## Supplementary Materials

The following supporting information can be downloaded at https://www.mdpi.com/article/10.3390/children13020178/s1, File S1: Preferred Reporting Items for Systematic Reviews and Meta-Analyses (PRISMA) 2020 checklist; File S2: Preferred Reporting Items for Systematic Reviews and Meta-Analyses (PRISMA) 2020 abstract checklist; Table S1: Adapted version of the Newcastle–Ottawa Assessment scale used in the present study for quality assessment (maximum 9 stars); Table S2: Details on quality assessment indices for the retrieved studies from adapted version of the Newcastle–Ottawa Assessment scale.

## Abbreviations

The following abbreviations are used in this manuscript:

MD	Moral development
ADHD	Attention-Deficit/Hyperactivity Disorder
PRISMA	Preferred Reporting Items for Systematic Reviews and Meta-Analyses
PICO	Population, Intervention/Exposure, Comparison, Outcome
NOS	Newcastle–Ottawa Scale
NEET	Not in Education, Employment, or Training
CU	Callous–unemotional
ODD	Oppositional defiant disorder
CD	Conduct disorder
